# Broadband THz to NIR up-converter for photon-type THz imaging

**DOI:** 10.1038/s41467-019-11465-6

**Published:** 2019-08-05

**Authors:** Peng Bai, Yueheng Zhang, Tianmeng Wang, Zhanglong Fu, Dixiang Shao, Ziping Li, Wenjian Wan, Hua Li, Juncheng Cao, Xuguang Guo, Wenzhong Shen

**Affiliations:** 1 Key Laboratory of Artificial Structures and Quantum Control, School of Physics and Astronomy, Shanghai Jiao Tong University, 200240 Shanghai, China; 2 Collaborative Innovation Center of Advanced Microstructures, 210093 Nanjing, China; 3 Department of Chemical and Biological Engineering, Rensselaer Polytechnic Institute, Troy, NY 12180 USA; 4 Key Laboratory of Terahertz Solid-State Technology, Shanghai Institute of Microsystem and Information Technology, Chinese Academy of Sciences, 200050 Shanghai, China; 5 School of Optical-Electrical and Computer Engineering, University of Shanghai for Science and Technology, 200093 Shanghai, China

**Keywords:** Optoelectronic devices and components, Terahertz optics

## Abstract

High performance terahertz imaging devices have drawn wide attention due to their significant application in healthcare, security of food and medicine, and nondestructive inspection, as well as national security applications. Here we demonstrate a broadband terahertz photon-type up-conversion imaging device, operating around the liquid helium temperature, based on the gallium arsenide homojunction interfacial workfunction internal photoemission (HIWIP)-detector-LED up-converter and silicon CCD. Such an imaging device achieves broadband response in 4.2–20 THz and can absorb the normal incident light. The peak responsivity is 0.5 AW^−1^. The light emitting diode leads to a 72.5% external quantum efficiency improvement compared with the one widely used in conventional up-conversion devices. A peak up-conversion efficiency of 1.14 × 10^−2^ is realized and the optimal noise equivalent power is 29.1 pWHz^−1/2^. The up-conversion imaging for a 1000 K blackbody pin-hole is demonstrated. This work provides a different imaging scheme in the terahertz band.

## Abstract (web summary)

Designing high performance THz pixelless imaging devices is nowadays challenging due to the inability to obtain normal incidence excitation using a grating coupler. Here, the authors report a broadband GaAs HIWIP-LED THz-NIR up-conversion imaging device avoiding the use of any grating coupler.

## Introduction

Terahertz (THz) imaging devices have attracted more attention and been intensively explored during the last two decades thanks to its numerous applications in healthcare, security of food and medicine, nondestructive inspection, scientific research, as well as national security application^1–6^. The imaging can be realized in pixel-based array imaging (e.g., linear array and focal plane array (FPA)) and the pixelless imaging mode. At present, FPA is still the most often used type.

The standard planar hybrid architecture, commonly used in near and mid-infrared FPA, is also expected to extend to THz region^7^. After years of development, the uncooled operated and low noise equivalent power (NEP) a-Si and VOx micro-bolometer commercial THz imaging cameras were realized by CEA-Leti and INO^8^. However, the performance of thermal detector is limited by a tradeoff between speed and sensitivity in many applications^9^. To acquire higher sensitivity, the frame rate for the commercial THz cameras is only 25 Hz and 50 Hz from CEA-Leti and INO, respectively^10,11^. This rate is enough for real time imaging but far away from the application of high speed detection. In addition, the response range of the reported micro-bolometer based THz cameras is 0.1–4 THz, which may be not broad enough for more high frequency application. FET (field effect transistor)-based THz direct detection may be another promising way to realize THz imaging^8^. The biggest advantage of this kind of detector is that the fabrication process is totally compatible to the low-cost standard silicon microelectronics processes technology. Similar to micro-bolometers, FETs are also not broadband and fast enough. Ge-based long wavelength infrared (LWIR) FPA has been widely used in astronomy and cosmological observations^9^. However, its performance is rarely improved in recent years.

Gallium Arsenide (GaAs)-based photon-type FPAs (quantum well photodetector (QWP)-FPA) have a great success and achievement in mid-infrared and far-infrared region due to its advantages of high detection sensitivity, fast response speed, wide response range, as well as high damage threshold^12,13^. As an extension of the traditional infrared (IR) QWPs to the THz frequency, the realization of the THz QWP FPA is not easy as expected^13,14^. Firstly, for the relative longer wavelength detection, the optical coupling structure is hard to design for imaging application in THz region^15,16^. Furthermore, owing to the low activation energy (~10 meV), THz QWPs must be operated at low temperature (<20 K)^17^. The connection of hybrid GaAs-based FPA-readout integrated circuit (ROIC) imaging device will be destroyed by repeated heating and cooling because of the severe thermal mismatch between the GaAs and Si. The dead pixels increase with the temperature decreasing^18^.

Pixelless imaging based on the semiconductor up-conversion technique has attracted tremendous research effort and made great progress at near-infrared^19–21^ and mid-infrared^22–24^ region in past 20 years. This technique makes use of an entire large size of device cell to  image directly. There is no separate pixel element at all in optical receiving terminal part, which simplifies the fabrication process significantly. The entire image is transmitted in the detector, then is restored by light emitting diodes (LED) and is finally ‘seen’ by a Si charge coupled device (CCD). The greatest advantage of such pixelless imaging is that no ROIC is required. Therefore, it solves the failure of Si-ROIC at low temperature naturally, which is a critical problem for many THz FPA imaging devices. And the cost would be greatly reduced at the same time. THz pixelless imaging based on the integrated QWP and LED is recently demonstrated to exhibit a promising application potential^18^. However, the 45° edge coupled geometry was adopted to realize the optical coupling, which caused the imaging spot of the quantum cascade laser (QCL) elongated in one direction. A possible solution is using the grating geometry to realize the normal incidence, but the design and fabrication of the grating is a great challenge for THz QWP-LED. In addition, the wavelength dependence of diffraction makes the grating coupler unsuitable for broadband or multicolor detection. Therefore, this QWP-LED pixelless imaging device is far from optimization.

In this work, a novel THz up-conversion device is realized, which is based on the integrated p-type GaAs homojunction interfacial workfunction internal photoemission (HIWIP) detector and LED for pixelless imaging^25,26^. The choice of a HIWIP detector allows normal incidence excitation thus bypassing the need for a grating coupler required for n-QWIP-LED device^27^. In addition, the HIWIP-LED up-converter shows a broadband photoresponse (4.2–20 THz) in contrast to the QWP-LED, which makes it general enough to be applied in more situations. Both the HIWIP detector part and the LED part in the integrated device were measured and shown high performance. Then, a blackbody and a quantum cascade laser are up-converted to a near-IR image and signal separately. Finally, the modulation transfer function (MTF), the factor of signal to noise (FSNR) together with the noise equivalent power (NEP) are used to evaluate the pixelless imaging quality of the HIWIP-LED device. This work provides a new imaging scheme in terahertz band.

## Results

### Device structure and I–V characterization

**Fig. 1:**
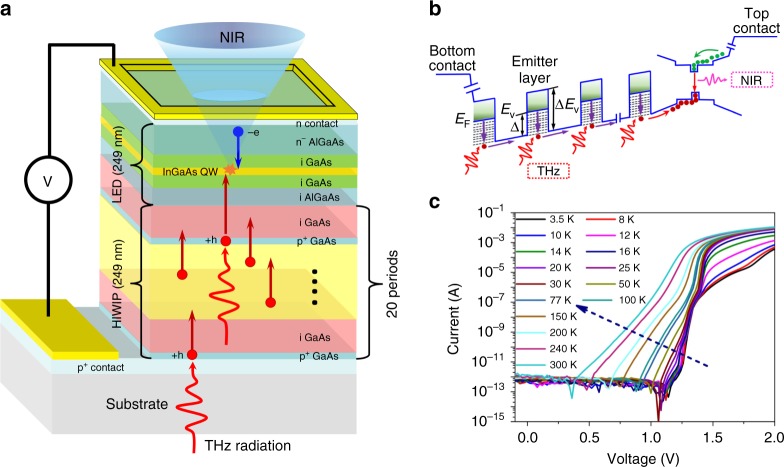
Device structure and detection mechanism of the integrated homojunction interfacial workfunction internal photoemission (HIWIP) detector and LED. **a** Structure of the HIWIP-LED up-converter device. **b** Band diagram of HIWIP-LED up-converter device. **c** The I–V characteristic (with 300 K background radiation) of the up-converter at different temperatures

The schematic structure of HIWIP-LED up-converter is shown in Fig. 1a. It is composed of a p-type GaAs HIWIP detector and a specially designed LED for low temperature, i.e. GaAs/AlGaAs double heterojunction LED inserted with InGaAs quantum well (AlGaAs/GaAs/InGaAs LED). The detailed parameters of the up-converter can be found in the Method section and Supplementary Note 5 (Supplementary Table 1). It also should be pointed that the connection layer between the HIWIP detector and the LED is intrinsic GaAs/AlGaAs layer without any doping, aiming to decrease the lateral diffusion of the photo-induced carriers and then avoid the image distortion^28^. Just like most of the other semiconductor up-conversion device, the HIWIP-LED device is a two terminal device, which means that we cannot adjust the bias voltage applied to the HIWIP part and the LED part separately^29^. The HIWIP-LED device could be seen as an up-converter in which a photoconductor connects in series with an LED. At a specific temperature, the influence of the HIWIP on the LED is only the change of the series resistance. When the HIWIP operating, photons at the THz band were absorbed by HIWIP detector first and transformed into photo-current under forward bias. The photon-generated carriers migrating to the active region of the LED recombine and emit in the near IR or the visible spectrum falling into the Si-CCD response rang. The extra energy for this up-conversion process is from the applied electric field.

The corresponding band diagram in Fig. 1b indicates the microscopic working mechanism of the up-conversion process. Free carrier absorption of the THz radiation occurs in the highly doped emitter layers followed by the internal photoemission of photo-excited carriers crossing the interfacial barrier under forward bias. Then the photo-excited carriers are injected into the active region of the LED, recombine and emit the near IR photons which are detected by the Si-CCD.

Figure 1c shows the I–V characteristic (with 300 K background radiation) of the up-converter at different temperatures. The I–V curves of the up-converter exhibit a turn-on behavior due to the diode structure in the LED^30^. This behavior requires the up-converter operates with the bias voltage higher than 1.4 V at the temperature below 20 K to ensure both the HIWIP part and LED part work under the optimum voltage and low background current. The dark current characteristic of the up-converter is given in Supplementary Note 1.

### Performance of the HIWIP

**Fig. 2:**
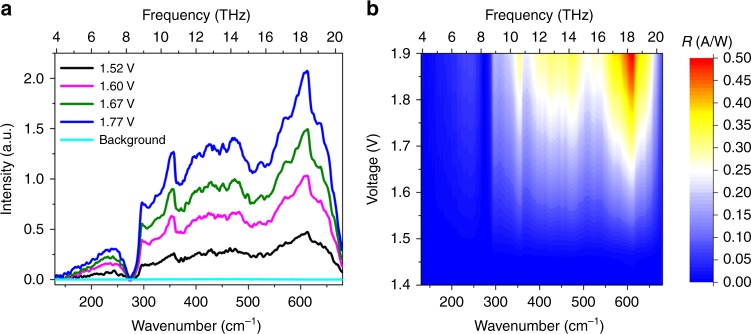
Photoresponse of the homojunction interfacial workfunction internal photoemission (HIWIP) detector in the up-converter. **a** Photocurrent spectra of the HIWIP-LED at 3.5 K under different bias voltages. **b** Responsivity of the HIWIP detector in the up-converter at 3.5 K under different bias voltages

The photocurrent spectrum at 3.5 K of the up-converter at different bias voltages is shown in Fig. 2a. This spectrum is identical with that of a single HIWIP detector if the threshold voltage is taken into account (the actual bias applied differs by 1.2 V)^27^. The spectrum shows a broadband photo-response from 150–680 cm^−1^ (4.2–20 THz) and the peak position is at ~18 THz. The deep valley between 270 and 300 cm^−1^ is corresponding to the transverse optical phonon energy in GaAs (Reststrahlen band).

The spectral response also shows a strong bias dependence, which increases significantly with increasing bias. However, the bias could not be increased infinitely as the dark current also increases with bias. The background in Fig. 2a means that there is no any photo-response when the bias voltage is below the threshold. The small valleys at the range from 300 cm^−1^ to 680 cm^−1^ are associated with the multiple phonons^31^.

The responsivity *R* of the up-converter exhibits an evident turn-on behavior that its value becomes valid when the bias voltage is greater than 1.4 V. This phenomenon is particularly evident in the mapping results of the *R* (as shown in Fig. 2b). The mapping results at 3.5 K give us much more  intuitive view of the dependence of the *R* on the bias voltage and wavenumber (or frequency). It’s obvious that the responsivity reaches the maximum (~0.5 AW^−1^) under the bias of 1.9 V at 18 THz.

### Performance of the LED

**Fig. 3:**
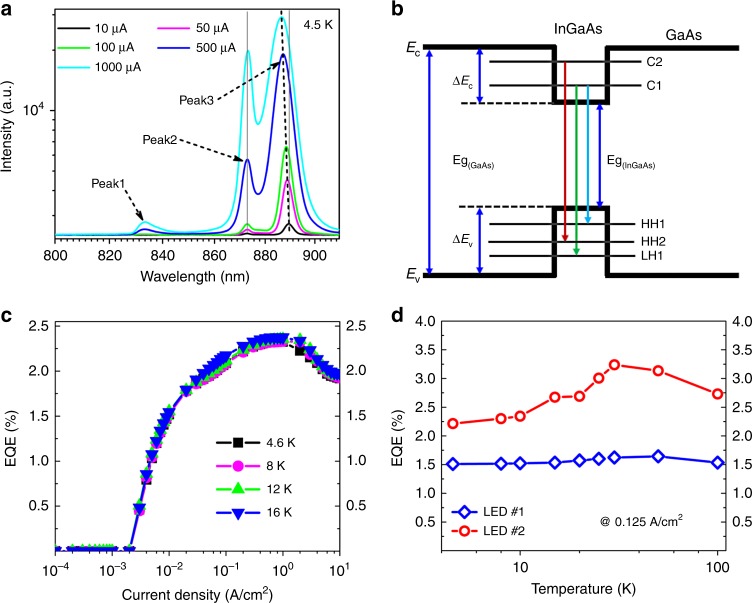
The luminescence spectra and external quantum efficiency of the LED part in the up-converter. **a** EL spectra of the HIWIP-LED at 4.5 K and at different  driving current. **b** Quantum well LED structure and interband transition. **c** The external quantum efficiency (EQE) of LED as a function of injection current density at low temperatures. **d** The external quantum efficiency (EQE) of the two independent LEDs at different temperatures with structure of InGaAs quantum well (#2) and conventional double heterojunction (#1), respectively

In the near-IR and mid-IR up-converters, double heterojunction GaAs/AlGaAs LED is usually used, which shows high quantum efficiency and a satisfactory effect. However, for the up-converter operated at low temperatures, such kind of LED may be not the best choice. We should adopt a LED specially designed for low temperature. The LED structure is an i-Al_0.2_Ga_0.98_As/GaAs/n-Al_*x*_Ga_1−*x*_As double heterojunction structure with a 9 nm intrinsic In_0.1_Ga_0.9_As quantum well insert in the center of the GaAs. The emission spectra of the LED part of up-converter at 4.5 K is displayed in Fig. 3a. There are three luminescence peaks at 833 nm (peak 1), 873 nm (peak 2), and 889 nm (peak 3), respectively. All of the luminescence peaks rise with the increasing of the driving current and the peak 3 presents a blue-shift effect. This blue-shift behavior is firstly observed in InGaAs quantum well LED, and similar effect exists in the GaAs diodes laser and InGaN/AlGaN double heterostructure LED, which is known as the band filling effect^32,33^. It should be noted that the luminescence from the intrinsic recombination of the GaAs material is at the wavelength of 816 nm (corresponding to the GaAs bandgap of 1.519 eV). The three peaks shown in Fig. 3a are all above the wavelength of 816 nm, which indicates the three luminescence peaks are all originated from the InGaAs quantum well.

To better understand the luminescent property of the InGaAs quantum well LED, the band structure of the In_0.1_Ga_0.9_As/GaAs quantum well is calculated by self-consistent solving the Schrodinger equation^34^. The plane wave expansion (PWE) method is used for accurate calculation and the lattice mismatch caused strain and bias caused Stark shift are neglected. The detailed calculation is given in the Supplementary Note 3.

Due to adequate consideration about the complexity of the valence band, the self-consistent solution of the calculation agrees well with experiment results. The peak 3 (889 nm) is due to the first conduction-subband to the first heavy hole subband transition (*C*_1_→*HH*_1_(885.79 nm), the peak 2 (873 nm) is resulted from the first conduction-subband to the first light hole subband transition (*C*_1_→*LH*_1_(876.88 nm) and the peak 1 (833 nm) originates from the second conduction-subband to the second heavy hole subband transition (*C*_2_→*HH*_2_(838.97 nm), respectively (as shown in Fig. 3b). The calculated result could also explain the injection current dependent luminescence intensity in Fig. 3a. With a low-level injection current, the *C*_1_→*HH*_1_ transition  dominates the luminescence and the peak 3 shows a stronger signal than the others. As the increasing of the injection current, the band filling effect occurs and the luminescence of peak 3 tends to saturation. Meanwhile, the intensity of peak 2 increases as the increasing of the injection current. The transition probability of *C*_2_→*HH*_2_ is much less than the other two transitions because most of the injection carriers occupy the ground state first.

Since the device usually works at low temperatures (<20 K), we pay more attention to the performance of LED at low temperatures. The external quantum efficiency (EQE) at the temperatures below 20 K are presented in the Fig. 3c. It is obvious that the EQE at different temperature is almost the same, which benefits from the significant non-radiative recombination suppression of the present LED. The EQE increases quickly when the injection current density is higher than 0.001 Acm^−2^. This behavior associates with the turn-on behavior of the HWIP-LED device. The external efficiency reached its maximum from 0.1 Acm^−2^ to 1 Acm^−2^, which is a trade-off result of the radiative recombination, non-radiative recombination and injection efficiency^35^. When the injection current density is higher than 1 Acm^−2^, the EQE displays efficiency drop, which is mainly caused by the severe non-radiative recombination and the carrier overflow. The corresponding maximum internal quantum efficiencies (*η*_i_) of the LED are all above 95% at the temperatures below 20 K. The calculation of internal quantum efficiency is presented in the Supplementary Note 3. Neglecting the influence of temperature, the calculated light extraction efficiency (LEE) is about 2.4% from the relation of $\eta _{{\mathrm{LED}}} = \eta _{\mathrm{i}} \cdot {\mathrm{LEE}} \cdot h\nu /e$ by numerically solving the rate equation^36^. At very low temperatures, the peak up-conversion efficiency *η*_up_ = 1.14 × 10^−2^ is thereout obtained at 18 THz under 1.9 V in terms of $\eta _{{\mathrm{up}}} = R \cdot \eta _{\mathrm{i}} \cdot {\mathrm{LEE}}\cdot h\nu /e$, which will be much larger as the bias increases.

To better understand the superiority of the LED structure specially designed in this study, two independent LED devices were fabricated. The external quantum efficiency of two LED samples as a function of the injected current density at the temperature below 100 K is shown in Fig. 3d. LED #2 has the same quantum well structure as the LED part in HIWIP-LED devices. LED #1 is the ordinary double hetero-junction GaAs/AlGaAs LED, which was widely used in previous up-conversion devices^20^. Apparently, LED #2 is better than LED #1 at low temperatures. The average external quantum efficiency of the LED #2 is about 72.5% higher than that of LED #1. The high efficiency of the quantum well LED is mainly due to the In_0.1_Ga_0.9_As quantum well structure. This kind of LED structure can improve the carrier injection efficiency and confine more carriers into the In_0.1_Ga_0.9_As quantum well, thereby promoting the radiation recombination efficiency. More importantly, the greatest advantage of the In_0.1_Ga_0.9_As well is that the emitted light is not absorbed by any other layers again^23^. This characteristic could not only improve the emitting efficiency, but also improve the imaging quality.

### Optical up-conversion and pixelless imaging

**Fig. 4:**
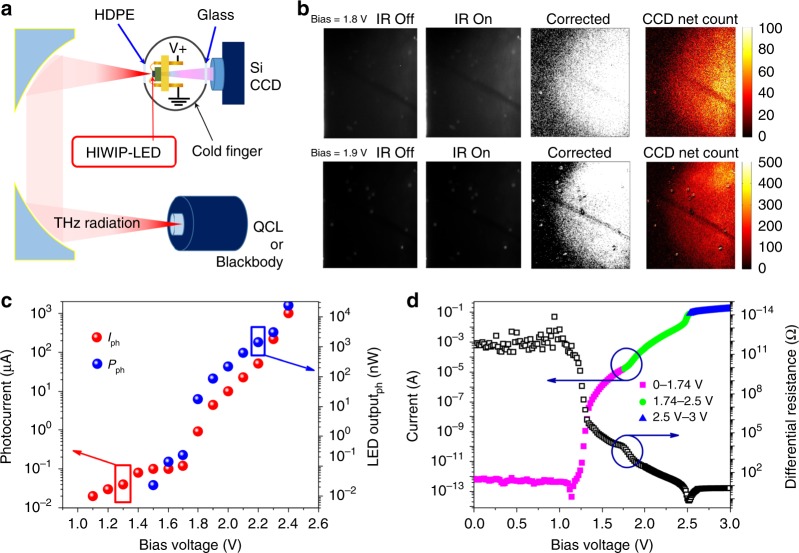
The direct up-conversion measurement using blackbody and quantum cascade laser (QCL) as the light source. **a** Optical setup for up-conversion measurement. **b** The up-converted image of the 0.025-inch-hole blackbody source (1000 K), the IR off and IR on means the device with and without IR illumination. **c** The laser induced net photocurrent and LED emitted up-converted net power as a function of bias voltage (i.e., the background was subtracted) at 3.5 K. **d** The I–V characteristic with 300 K background radiation of the up-converter and corresponding differential resistance at 3.5 K

The optical setup of the up-conversion pixelless imaging is presented in Fig. 4a. Radiation from the blackbody or QCL is collimated and focused by a pair of off-axis parabolic mirrors. The focal length of the off-axis parabolic mirror is 10 cm and the distance of the two off-axis parabolic mirrors is 25 cm. The HIWIP-LED sample is placed at the focal position in the liquid helium cryostat. The front window of the cryostat is high density polythene to avoid the near-infrared or visible radiation influence. Whereas the rear window is quartz window to allow the transmission of the near-infrared emission light. The emitted image from the backside of the up-converter is detected by a Si CCD camera. The CCD is set very close to the window of the cryostat and the distance between the HIWIP-LED and the lens of the CCD camera is about 6 cm. The focal point of the CCD  is adjusted align to the position of the HIWIP-LED when measured. Figure 4b displays the up-converted image of the 0.025-inch-hole blackbody source (1000 K) at 1.8 V and 1.9 V, respectively. IR off and IR on means the blackbody source illumination is turned off and on.

In order to obtain the target image, the LED emission originate from the background current is subtracted, just like the method used in many imaging devices such as conventional FPA^22–24^. It’s clear that there is an obvious circle image in the background corrected picture. The image at 1.9 V is better than that at 1.8 V. The corresponding CCD net count are also given in Fig. 4b to reveal the signal strength. The dark or bright spots on the picture is known as the LED emission hot spot at low temperatures with low injection level^37^. The present image is not a perfect circle because the actual effective area of the device is 700 × 700 μm^2^ by deducting the top ring electrode, which is only a little larger than the diameter of the blackbody source hole (635 μm).

A home-made monochromatic QCL source with a lasing frequency of 4.26 THz is also used to measure the up-conversion performance. The peak continuous wave lasing power of the QCL is about 3 mW with the injection current of 0.7 A. The characteristics of the QCL are presented in the Supplementary Note 2. The induced net photocurrent and LED emitted up-converted power (i.e., the background was subtracted) are shown in Fig. 4c. There exists an apparent photocurrent in the device even though the laser frequency is close to the cutoff frequency of the HIWIP detector (Fig. 2a) where the responsivity is extremely low (<0.001 AW^−1^). The net photocurrent and emitted power increase suddenly at about 1.7 V. The luminescence between 1.4 V to 1.7 V (at low temperature and low driving current) is caused by the hot spot in the device. The hot spot is caused by the material defects or surface imperfections^37^. Meanwhile, normal LED luminescence (not caused by hot spot) happens between 1.74–2.5 V, which agrees with Fig. 4d, where the I–V characteristic with 300 K background radiation and the corresponding differential resistance at 3.5 K is given. Figure 4c, d indicate a proper operation bias window of 1.74–2.5 V. In this region, the response in the HIWIP part is high (high photocurrent and thereby the low differential resistance) and the current resulted from the background is also suppressed within an acceptable range. It should be noted that both the photocurrent and up-conversion induced LED power get to a much higher value at high bias, which is due to the high electrical field induced impact ionization^38^ and hot carrier injection effect^39^.

### Evaluation of imaging quality

The advantage of the HIWIP-LED device is high broadband photoresponse and it allows normal incidence absorption of the light benefiting from the detection mechanism of the HIWIP detector. These features make it promising to realize a broadband pixelless imaging device. For a typical imaging device, the spatial resolution, signal-to-noise ratio and NEP  are important for practical application.

The modulation transfer function (MTF) is employed to evaluate the image conversion characteristics of the HIWIP-LED up-converter^28^, which represents the ratio of the image contrast to the object contrast:

$$\begin{array}{*{20}{l}} {{\mathrm{MTF}}\left( f \right)} \hfill & = \hfill & {p_c\exp \left( { - 4\pi ^2l^2f^2} \right)} \hfill \\ {} \hfill & {} \hfill & { \times \frac{{1 - \left( {1 - p_c} \right)^N\exp \left( { - 4\pi ^2l^2f^2N} \right)}}{{1 - \left( {1 - p_c} \right)\exp \left( { - 4\pi ^2l^2f^2} \right)}}} \hfill \\ {} \hfill & {} \hfill & { \times \frac{{1 - \sigma \eta _{\mathrm{i}}\left( {\alpha _1/\alpha _2} \right)}}{{1 + 4\pi ^2l_d^2f^2 - \sigma \eta _{\mathrm{i}}\left[ {\alpha _1\alpha _2/\left( {\alpha _2^2 + 4\pi ^2f^2} \right)} \right]}}.} \hfill \end{array}$$

Where *p*_*c*_ is the capture probability of holes in the GaAs emitter layers, *l* is characteristic diffusion length of emitter layer, *N* is the period number of the emitter/intrinsic layers, *l*_*d*_ is the diffusion length of carriers in the LED active layer, *σ* is the fraction of the NIR photons trapped in the LED due to the total internal reflection, *η*_*i*_ is the internal quantum efficiency of the LED, *α*_*1*_ and *α*_*2*_ represent the effective absorption coefficient of the active layer and the average the absorption coefficient of LED^28^. *f* is the spatial frequency. An object can be looked upon as the spatial distribution of the intensity and color of light. If it is considered to be composed of lines of various pitches in various directions, the spatial frequency *f* reflects the characteristics of this distribution and is the reciprocal of structural size with a unit of line pairs per millimeter (lp/mm). Due to the limitation of the diffraction limit, for an imaging system with specific wavelength (*λ*), images with spatial frequencies greater than *f*_λ_ cannot be resolved, i.e. $f \le f_\lambda = \frac{1}{\lambda }$ should be satisfied. Therefore, the dependence of MTF on *f* reflects the imaging quality at different spatial resolution in fact.

The operation mechanism of HIWIP-LED involves of three processes. First of all, the incident light is absorbed by the HIWIP part. Then, the photo-generated carriers transport from the HIWIP part to the active region of the LED. Finally, the radiation recombination occur in the quantum well of the LED and emit near-infrared photons. The MTF is deduced from solving the Poisson equation and current continuity equation, where the three processes were taken into consideration (see Supplementary Note 3 for detailed simulation). Therefore, the MTF describes the ability of the HIWIP-LED to transfer the object contrast to the image contrast.

**Fig. 5:**
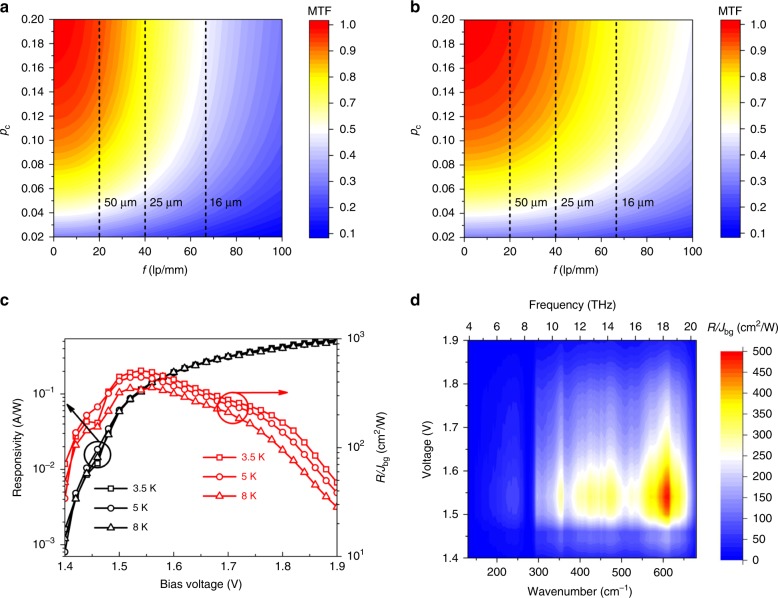
Imaging quality evaluation using the modulation transfer function (MTF) and factor of signal to noise (FSNR) characterized by *R/J*_bg_. **a** The modulation transfer function (MTF) of the HIWIP-LED device with the double heterojunction LED (LED #1). **b** The modulation transfer function (MTF) of the HIWIP-LED device with the InGaAs quantum well LED (LED #2). **c** Peak responsivity and the corresponding values of *R/J*_bg_ of the HIWIP-LED at  low temperatures under different bias voltages. **d** The mapping result of *R/J*_bg_ as a function of bias and wavenumbers at 3.5 K

Figure 5a, b show the calculated variation of the MTF with *p*_*c*_ and *f* for the up-converter with double heterojunction GaAs/AlGaAs LED (LED #1) and InGaAs quantum well LED (LED #2), respectively. Both of the structures show that the MTF increases with the capture probability (*p*_*c*_) increasing, which seems to be an anomaly phenomenon. The increase in *p*_*c*_ means that the photocurrent density injected to the LED will decrease. However, it has been proved that the signal photocurrent decreases much slowly compared with  the dark current or background photocurrent, which leads to the enhancement of the image contrast^28^. This characteristic reveals that a better imaging quality means some sacrifice of the HIWIP detector response. The dash lines indicate the limit *f*_*λ*_ for a given incident light. It is noted that the MTF of the up-converter with LED #2 is much better than that of the up-converter with LED #1. In contrast to the device with LED #1, the MTF of the up-converter with LED #2 is almost larger than 0.5 in the whole range of spatial frequency we studied, which means the image perceives sharpness and resolution even in size of details. This is because the emitted light of the quantum well LED will not be absorbed again by any other layer, which would not cause remarkable image distortion. From this point of view, the InGaAs quantum well could not only improve the up-conversion efficiency, but also improve imaging quality.

The imaging quality can also be evaluated by the factor of the signal to noise (FSNR) of the HIWIP-LED device. FSNR is characterized by the ratio of *R*/*J*_bg_^18^, in which *R* is the calibrated responsivity and the *J*_bg_ is the background current density. The calculated FSNR and responsivity  are shown in Fig. 5c. FSNR can be viewed and treated as the signal to noise ratio when 1 cm^2^ area of the device is illuminated by 1 watt light. It’s clear that the FSNR does not increase monotonously with the bias in contrast to the responsivity. The FSNR are nearly all above 100 at the operation voltage (1.45–1.8 V) and the maximum FSNR is about 500 at ~1.5 V. The results show that the proper operating bias voltage for high imaging quality located in the region of 1.5–1.6 V, where the FSNR gets its peak value. The reason is that the FSNR is determined by *R* together with *J*_bg_ and it’s a tradeoff of *R* and *J*_bg_. High operation voltage is helpful to increase *R*, nevertheless, the dark current will also increase enormously, leading to the decrease of FSNR. Figure 5c, d indicate the best FSNR does not correspond to the maximum responsivity but the maximum signal to noise ratio, which is in good agreement with calculated results of MTF. When the voltage is set to be 1.8 V, the FSNR becomes one half of the peak value. But the signal strength at 1.8 V is 2 orders higher than that at 1.5 V (shown as the net output power shown in Fig. 4c), which exactly explains why we recognize the up-conversion image at 1.8 V instead of 1.5 V.

It is noted that the FSNR also exhibits a turn-on behavior when the bias voltage greater than 1.4 V. This phenomenon is particularly obvious in the mapping results of FSNR shown in Fig. 5d. The mapping results at 3.5 K give a much more  intuitive  view of the dependence of the FSNR on the bias voltage and wavenumber (or frequency).In a wide response range (from 6–21 THz), the FSNR of this kind of HIWIP-LED up-converter is always larger than 100 when the operation bias is in the range of 1.5–1.8 V at 3.5 K, showing a relatively ideal imaging quality. This means that the imaging quality could be guaranteed over a wide range of wavelength. In addition, according to Fig. 4c, d, the best working voltage region is 1.74–2.5 V. With an overall consideration of signal intensity, the background current and the imaging quality discussed above, we find the optimum working voltage of the up-converter is 1.8–1.9 V.

**Fig. 6:**
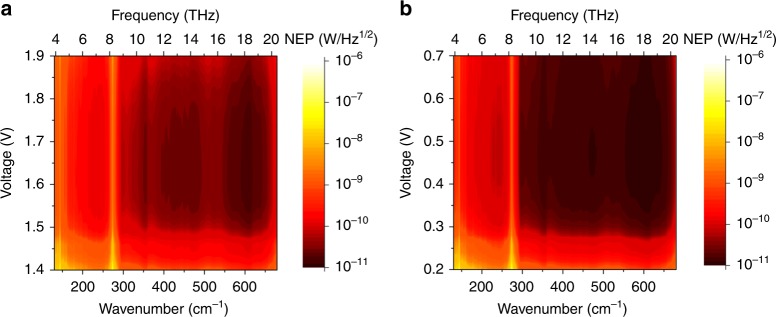
Calculated noise equivalent power (NEP) of the up-conversion imaging system and the single homojunction interfacial workfunction internal photoemission (HIWIP) detector. **a** NEP of the up-conversion imaging system under different bias voltage at 3.5 K. **b** NEP of the single HIWIP detector with the exactly same parameters as the detector part in HIWIP-LED up-converter under different bias voltage at 3.5 K

The noise equivalent power (NEP) is a figure of merit for photodetectors. Figure 6a, b show the calculated NEP under different bias voltages at 3.5 K for the up-conversion imaging system and single HIWIP detector, respectively^40,41^. The detailed calculation of the NEP is given in the Supplementary Note 3. The voltage difference for the two figures is due to the turn on voltage of HIWIP-LED. We can find that the NEP for all frequencies are almost below the level of 100 pWHz^−1/2^ and the optimal value is 29.1 pWHz^−1/2^ at 600 cm^−1^ between 1.5–1.9 V. In contrast, the optimal NEP for single HIWIP is 12.4 pWHz^−1/2^. The NEP of the HIWIP-LED is slightly larger than that of the single HIWP detector in the whole photosensitive region, indicating that effect of the noise from the extra introduced up-conversion process does exist. It should be noted that the degradation of NEP is mainly due to the relative lower LED light extraction efficiency. Nevertheless, we think that the NEP of HIWIP-LED imaging system could be further improved if proper coupling between the HIWIP-LED up-converter and Si CCD is adopted, e.g., by means of optical adhesive. If the thickness of the optical adhesive is comparable to the wavelength of the photon emitted by the LED, the photon tunneling effect will occur which can lead to a much higher light extraction efficiency. Theoretical value of the light extraction efficiency caused by photon tunneling effect can reach up to 100%. As high as 81% of light extraction efficiency has been achieved experimentally^42^. Even though it is slightly higher than that of the single HIWIP detector, NEP of the overall up-conversion imaging device is still comparable and competitive among the state of the art THz imaging devices.

## Discussion

In conclusion, we have proposed a novel broadband THz up-conversion photon-type imaging device operating at the range of 4.2–20 THz based on the HIWIP-LED up-converter and Si CCD. In contrast to QWP-LED, the HIWIP-LED could achieve broadband photo-response and absorb the normal incident light without any grating coupler. The overall comparison between the QWP and HIWIP is given in Supplementary Note 4. A peak responsivity for the HIWIP detector of 0.5 AW^−1^ was achieved at 18 THz. Aiming at low operation temperature and low driving current, the luminescence property for the specially designed LED was measured and analyzed systematically. The average external quantum efficiency of the LED adopted in the up-converter resulted in a 72.5% improvement at low temperatures in contrast to the ordinary double hetero-junction GaAs/AlGaAs LED widely used in previous up-conversion devices. A peak up-conversion efficiency of 1.14 × 10^−2^ was obtained. The THz HIWIP-LED up-converter based pixelless imaging for a 1000 K blackbody pin-hole was realized. Moreover, the 4.26 THz QCL signal could also be up-converted to a NIR signal effectively. The minimum noise equivalent power (NEP) of the HIWIP-LED up-conversion imaging system is 29.1 pWHz^−1/2^. The imaging performance of the HIWIP-LED was analyzed using MTF and NEP, which reflect the superiority of the HIWIP-LED. This work provides a new imaging scheme for terahertz band.

Thanks to the wide photo-response coverage (4.2–20 THz), the proposed HIWIP-LED up-conversion photon-type imaging device is potential and promising in many applications. First of all, there are some explosives that the feature band are around or higher than 5 THz, such as TNT (5.6, 8.2, 9.1, 9.9 THz)^43,44^ and NH_4_NO_3_ (4, 7 THz)^45^. So it could be used in security area for explosive detection. Secondly the feature band central position frequency of some drugs also around or higher than 5 THz such as Acetaminophen (6.5 THz) and Naproxen sodium (5.2, 6.5 THz)^46^, which makes it potential in healthcare and medicine security. Moreover, the broadband frequency response range corresponding to the far-infrared region, which is the exactly in the FIR region of the astronomical observation. For example, the Spitzer Space Telescope (launched in August 2003) and the James Webb Space Telescope (JWST, delayed to launch after 2020) were designed to detect the wavelength of 5–38 μm (7.9–60 THz) and 5–28 μm (10.7–60 THz)^9^.

In addition, one of the most important advantage of the GaAs-based photon-type detector is that it has high speed/frequency capability and there are no competitive alternatives in IR/THz region^12^. Thus the broadband THz HIWIP-LED up-converter based imaging in this work may be promising in the application of high speed THz imaging applications.

## Methods

### The wafer details of the up-converter

The HIWIP-LED up-converter consists of a p-GaAs HIWIP broadband THz detector and an AlGaAs/GaAs/InGaAs quantum well LED directly grown by MBE on 600 μm thick semi-insulating GaAs substrates. The p-type HIWIP detector consists of a 300 nm thick bottom contact layer doped with 3 × 10^18^ cm^−3^ Be, a 100 nm bottom emitter layer doped with 8 × 10^18^ cm^−3^ Be and 20 repeats of p-GaAs/i-GaAs (emitter layer/intrinsic layer) layers with emitter layer and intrinsic layer thickness of 80 nm and 15 nm. The p-GaAs emitter layer is doped with 8 × 10^18^ cm^−3^ Be to realize a high internal photon emission efficiency. The period number of the emitter/intrinsic layer is optimized with fully consideration of the ionization effect^38^. The LED structure is an i-Al_0.2_Ga_0.98_As/GaAs/n-Al_*x*_Ga_1–*x*_As double heterojunction structure with a 9 nm intrinsic In_0.1_Ga_0.9_As quantum well insert in the center of the GaAs. The thickness of the GaAs and Al_x_Ga_1-x_As on both sides of the In_0.1_Ga_0.9_As quantum well is 40 nm and 80 nm, respectively. The n-Al_x_Ga_1-x_As adopted an aluminum component grading layer (*x* = 0.02→0.1) and doped with Si to 2.5 × 10^18^ cm^−3^. An intuitive table of the detailed parameters are presented in the Supplementary Note 5. The top of device is 50 nm n-GaAs doped with Si to 2.5 × 10^18^ cm^−3^ for n-contact. The samples were fabricated using standard photo lithographic techniques. The top electrical connection is a narrow ring contact formed by deposition of PdGe/Ti/Pt/Au using electron beam evaporation. And the bottom p-contact is made by Ti/Pt/Au. The mesa is 1000 × 1000 μm^2^. Subtracting the top ring contact, the effective detection area is 700 × 700 μm^2^. The samples were mounted on 14 pin packages for electrical and optical measurements.

### Measurement details

All measurements were carried out at low temperatures. The photocurrent spectra at 3.5 K and different bias voltages shown in Fig. 2a are measured using a Fourier transform infrared spectrometer (Bruker VERTEX 80 IFS 66 v/s). The responsivities of the HIWIP-LED at different bias voltages shown in Fig. 2b are acquired using a calibrated blackbody (Infrared Systems Development Corporation IR-564/301) together with a low noise current preamplifier (Model SR570) and a lock-in amplifier (Model SR830). The LED’s emission spectrum with different driving current in Fig. 3a were measured using a fiber spectrometer (Ocean optics QE65PRO). The optical fiber probe was set close to the glass window of the cryostat to acquire the emission spectrum. EQE of LED in Fig. 3c, d were calculated from the driving current and emission light power. The emission light power is measured by Thorlabs S130C large area Si slim photodiode. The emission power at low temperatures were calibrated with a calibration coefficient, which was obtained at the room temperatures. Firstly, the large area Si photodiode close to the HIWIP-LED device to measure the emission power (*P*_out-c_). Then, place the HIWIP-LED inside the cryostat, using the large area Si photodiode close to the glass window of the cryostat to measure the emission power (*P*_in-c_). The calibration coefficient was determined by comparing the *P*_out-c_ and *P*_in-c_. The CCD used for imaging setup is a commercial Andor Si CCD camera (iKon-M 934 BR-DD).

## Supplementary information

Supplementary Information

## Data Availability

The data that support the findings of this study are available from the authors on reasonable request, see author contributions for specific data sets.
