# Supplementary material for: Broadband THz to NIR up-converter for photon-type THz imaging
Source: Nat Commun. 2019 Aug 5;10:3513. doi: 10.1038/s41467-019-11465-6 (PMC6683120; doi:10.1038/s41467-019-11465-6)
Supplement: Supplementary file 1 — Supplementary Information [file 41467_2019_11465_MOESM1_ESM.pdf]

Supplementary Information for

Broadband THz to NIR up-converter for photon-type THz imaging

Peng Bai, Yueheng Zhang, Tianmeng Wang, Zhanglong Fu, Dixiang Shao, Ziping Li,

Wenjian Wan, Hua Li, Juncheng Cao, Xuguang Guo, Wenzhong She

Supplementary Note 1: Dark current

The dark current as a function of bias voltage of HIWIP-LED at different temperatures was shown in Supplementary Figure 1

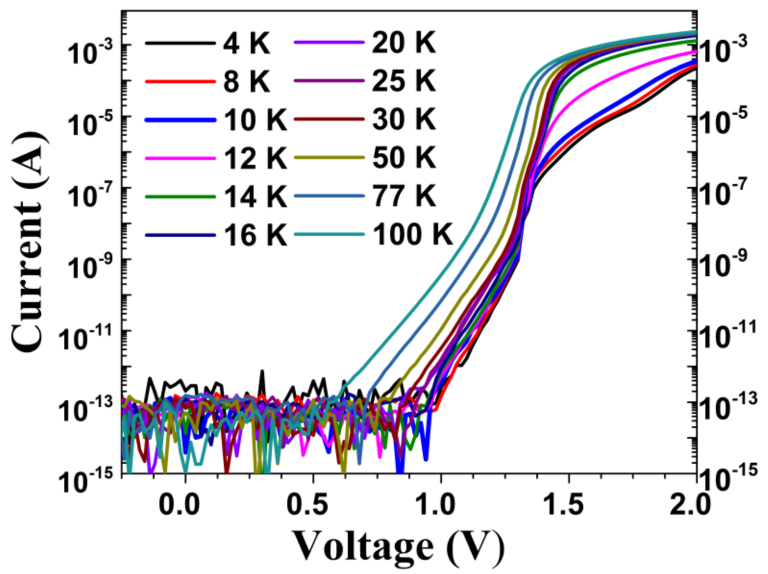

Supplementary Figure 1. The dark current as a function of bias voltage of the up-converter at different temperatures.

We can find that the up-converter exhibit a turn-on behavior due to the diode structure. And the dark current increases rapidly as the temperature increases. The current increase is mainly from HIWIP part contribution. The dark current for the HIWIP detector is the sum of thermionic emission (TE) current, thermionic field emission (TFE) current and field emission (FE) current. At low temperatures, the FE current dominates, which caused a relative lower dark current. The TE current and TFE current are especially sensitive to the temperature, which caused the rapid increase of the dark current for the detector with the temperature increasing. In order to acquire better signal-to-noise ratio and imaging quality, all the measurements were carried out at about liquid helium temperature or lower (3.5 K).

Supplementary Note 2: Characteristic of QCL

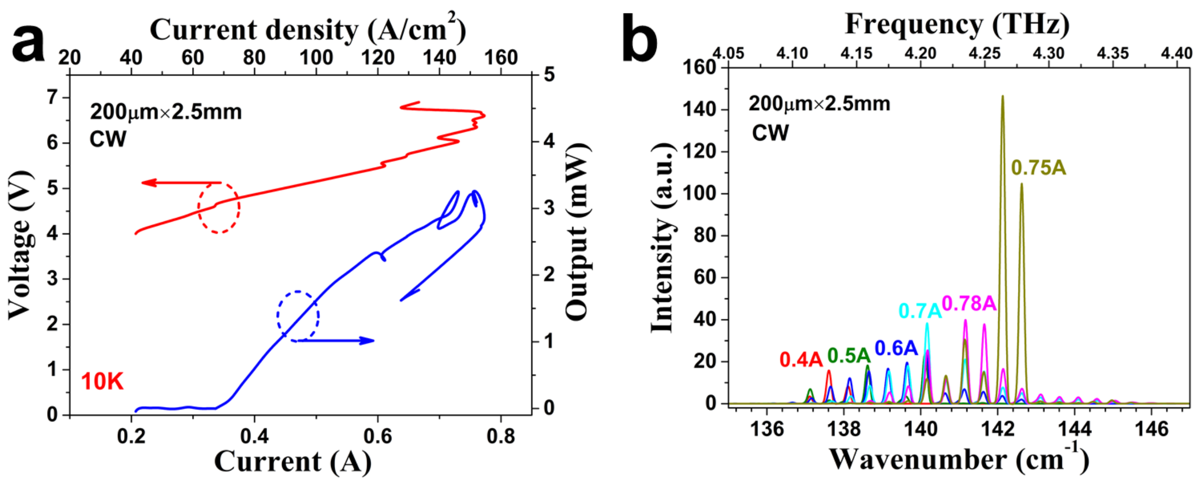

Supplementary Figure 2. Characteristic of the quantum cascade laser. (a) Output power of the QCL as a function of current, (b) laser spectrum of QCL.

Supplementary Note 3: Simulation details

We simulated the internal quantum efficiency of the $In_{0.1}Ga_{0.9}As$ quantum well LED (IQE), the band structure of the LED, modulation transfer function (MTF) as well as the noise equivalent power (NEP) of the HIWIP-LED. Details on these three simulation work are as follows.

IQE of the $In_{0.1}Ga_{0.9}As$ quantum well LED

To better understand the superiority of the QW-LED at low temperatures and the severe efficiency droop at high temperatures, we should make clear of its recombination mechanism inside of the device at different temperatures. The non-radiative Shockley-Read-Hall (SRH) recombination, radiative recombination, and Auger recombination together determine the luminescence property of the LED. The ABC model is a simple and widely used approach to study these three principal channels of recombination in LED [1]. The temperature dependent recombination mechanism of the LED will be much clearer if the SHR (A), radiative (B), and Auger recombination (C) coefficients at all temperatures were given. Thus the most direct way to study the LED performance is to measure the

three coefficients at different temperatures. However, it's impractical and difficult to realize the measurement at such an extreme low temperature directly (~ 4 K).

A developed ABC model provides a way to analyze the LED performance with the peak point of the internal quantum efficiency (IQE) curve for the only fit parameter. The injection current dependence of the LED IQE explicitly by numerically solving the equation [2]:

$$\eta_i = 1 - \frac{(1-\eta_{\max})}{2J}(1+\frac{\eta_i J}{\eta_{\max} J_{\max}})\sqrt{\frac{\eta_i J J_{\max}}{\eta_{\max}}}$$

Where η_i is the internal quantum efficiency (*IQE*), J is the injection current density, $\eta_{\max}$ and $J_{\max}$ is the maximum value of *IQE* and the corresponding injection current density. J_{max} is exactly known by the measurement of the current density dependent electroluminescence efficiency (*EQE*) and η_{max} can be fitted in terms of the measurement results. This method points that there exists only one *IQE* curve for given J_{max} and η_{max} values regardless of A, B, and C coefficients. The corresponding maximum internal efficiencies (η_i) of the LED are all above 95% at the temperatures below 20 K. Neglecting the influence of temperature, the calculated light extraction efficiency (*LEE*) is of about 2.4% from the relation of $\eta_{\mathrm{LED}} = \eta_i \cdot LEE \cdot h\nu / e$ by numerically solving the above equation.

Band structure of the LED

To better understand the luminescent property of the InGaAs quantum well LED, the band structure of the $In_{0.1}Ga_{0.9}As/GaAs$ quantum well is calculated by self-consistent solving the Schrodinger equation [3].

$$\left\{-\frac{\hbar^2}{2}\frac{\partial}{\partial z}\left[\frac{1}{m^*(z)}\frac{\partial}{\partial z}\right] + V_{\mathrm{QW}}(z) + V_{\mathrm{H}}(z) + V_{\mathrm{xc}}(z)\right\}\varphi(z) = \varepsilon\varphi(z)$$

Here, m^* is the electron effective mass, $\hbar$ is the reduced Planck constant, V_{QW} is the stepwise potential energy representing the conduction band offset profile, V_{H} is the Hartree potential energy

obtained from Poisson's equation, V_{xc} is the exchange-correlation potential energy which is given by the local density approximation based on the density functionaltheory, φ is z-direction envelope function, and ε is eigen-energy. The plane wave expansion (PWE) method is used for accurate calculation. The lattice mismatch caused strain and bias caused Stark shift is neglected. The band-gaps of GaAs and $In_{0.1}Ga_{0.9}As$ are taken as 1.518959eV and 1.36495 eV at 4 K respectively. The ratio of conduction and valence band offsets is set as 6:4. The effective masses of the well (m_w^*) and barrier (m_b^*) are ($m_w^* = 0.05873m_0, m_b^* = 0.0916m_0$) for electron, ($m_w^* = 0.5m_0, m_b^* = 0.51m_0$) for heavy hole and ($m_w^* = 0.0764m_0, m_b^* = 0.082m_0$) for light hole respectively with m_0 the electron mass. Detailed plane wave expansion (PWE) method to self-consistent solve the Schrodinger equation could be found in Ref [4].

Modulation transfer function (*MTF*) of the HIWIP-LED

Modulation transfer function (*MTF*) is an important performance index to evaluate the spatial resolution of the imaging device. The calculation of *MTF* for the HIWIP-LED device involves two steps: photo-current and the current-photon process. Similar to the QWP-LED device, the photo-current process of the HIWIP-LED was analyzed by solving the current continuity equation [5]:

$$\frac{\partial p}{\partial t} = D\left(\frac{\partial^2 p}{\partial x^2} + \frac{\partial^2 p}{\partial y^2}\right) - \frac{\partial (vp)}{\partial z} + \sum_{1}^{N}(-p_c vp + G\phi^{in})\delta(z - mL),$$

where p is the hole concentration in the continuum, D is the diffusion coefficient of the holes, p_c is the capture probability of holes in the GaAs emitter layers, so the quantity $p_c vp$ is the rate of the hole capture, and $L=we+wi$ is the thickness of one period. Since the thickness of the emitter layer we is usually much smaller than the thickness of the intrinsic layer wi (wi=80 nm), it is reasonable to employ the representation of the emitter layer by a δ function, just like the case of QWIP. N is the number of

periodic structure in the HIWIP FIR detector, *mL* represents the coordinate *z* of a certain emitter layer plane, in is the photon flux of the incoming THz light, $G\varphi^{in}$ is the rate of holes photoexcited across the barriers.

Similarly, the subsequent current-photon process of the HIWIP-LED was analyzed by solving the current continuity equation [6]:

$$-D_p \frac{d^2 \sum_{ph}}{dx^2} + (\frac{1}{\tau_r} + \frac{1}{\tau_n}) \sum_{ph} = \frac{j}{q} + v_g \alpha_1 \phi^{out}$$

Where D_p is the diffusion coefficient of the active layer of the LED, $\sum_{ph}$ is the areal density of the photo-generated holes, τ_r and τ_n is the lifetime of the radiative recombination and nonradiative recombination in the active layer, v_g is the group velocity of the photon, α_1 is the absorption coefficient of the active layer, ϕ^{out} is the photon flux density.

By solving the above equations, the *MTF* of the HIWIP-LED is:

$$\begin{aligned} MTF(f) &= p_c \exp(-4\pi^2 l^2 f^2) \\ &\times \frac{1-(1-p_c)^N \exp(-4\pi^2 l^2 f^2 N)}{1-(1-p_c)\exp(-4\pi^2 l^2 f^2)} \\ &\times \frac{1-\sigma\eta_i(\alpha_1/\alpha_2)}{1+4\pi^2 l_d^2 f^2 - \sigma\eta_i[\alpha_1\alpha_2/(\alpha_2^2+4\pi^2 f^2)]}. \end{aligned}$$

σ is the fraction of the NIR photons trapped in the LED due to the total internal reflection, η_i is the internal quantum efficiency of the LED, α_1 and α_2 represent the effective absorption coefficient of the active layer and the average the absorption coefficient of LED.

Noise Equivalent Power of the HIWIP-LED

The noise equivalent power (NEP) is a figure of merit for photodetectors. In practical application, the HIWIP-LED up-converter is used in association with a commercial Si-CCD. Thus the up-converter and the Si-CCD should be regard as a united imaging system when we evaluate the overall *NEP*.

According to a developed noise theory of the up-conversion detectors [12], the noise of the HIWIP-LED imaging system can be expressed as:

$$\begin{aligned} i_{\mathrm{n}}^{2} &= (\eta_{\mathrm{Si}}\eta_{\mathrm{LED}})^{2}(4eg_{\mathrm{HW}}i_{\mathrm{bg}}\Delta f + 4eg_{\mathrm{HW}}i_{\mathrm{dark}}\Delta f) \\ &+ (\eta_{\mathrm{Si}}\eta_{\mathrm{LED}})^{2}(2ei_{\mathrm{bg}}\Delta f + 2ei_{\mathrm{dark}}\Delta f) \\ &+ 2e\Delta f\,\eta_{\mathrm{Si}}\eta_{\mathrm{LED}}(i_{\mathrm{bg}} + i_{\mathrm{dark}}) + 2ei_{\mathrm{dark,Si}}\Delta f \end{aligned}$$

where η_{Si} is the quantum efficiency of the Si CCD at the luminescence wavelength of the LED; η_{LED} is the external quantum efficiency of the LED, which is determined by the internal quantum efficiency and the light extraction efficiency (currently ~ 2.4%) simultaneously; e is the elementary charge; g_{HW} is the gain in the HIWIP; i_{bg} is the background photocurrent of the 300 K radiation; $i_{\mathrm{dark,}}$ is the device dark current, Δf is the system measurement bandwidth; $i_{\mathrm{dark,}\,Si}$ is the dark current of the Si CCD. The first item of the above equation is mainly determined by the HIWIP detector, the second item is mainly determined by the LED, and the last two items are mainly determined by the Si CCD. The influence the additional conversion step on the noise could be considered from two aspects. It could increase a bit because of the introduction of LED. On the other hand, it would be decreased greatly because of the low light extraction efficiency. Therefore, the total noise is actually reduced. (the detailed theoretical explanation of the extra introduced noise from the introduction of LED can be found in another PD-LED type up-conversion detector[13]. The responsivity of the up-conversion imaging system is $R_{\mathrm{imag}} = R\eta_{\mathrm{LED}}\eta_{\mathrm{Si}}hc/\lambda_{\mathrm{out}}e$.

So, the *NEP* of the up-conversion imaging system is:

$$NEP = \frac{i_{\mathrm{n}}}{R_{\mathrm{imag}}} = \frac{e\lambda_{\mathrm{out}}}{hcR}[(4eg_{\mathrm{HW}}i_{\mathrm{bg}}\Delta f + 4eg_{\mathrm{HW}}i_{\mathrm{dark}}\Delta f) + (2ei_{\mathrm{bg}}\Delta f + 2ei_{\mathrm{dark}}\Delta f) + \frac{2e\Delta f}{\eta_{\mathrm{LED}}\eta_{\mathrm{Si}}}(i_{\mathrm{bg}} + i_{\mathrm{dark}}) + \frac{2ei_{\mathrm{dark,Si}}\Delta f}{(\eta_{\mathrm{LED}}\eta_{\mathrm{Si}})^2}]^{1/2}$$

For commercial Si CCD, the dark current can be suppress to an extreme low level, so the last item can be neglected.

Supplementary Note 4: Comparison between QWP and HIWIP

Here, we will also give the comparison of QWP and HIWIP on their detection mechanism and the situation of practical applications. The quantitative data of their performance will also be given.

The schematic diagram of detection mechanism of a QWP could be found in Supplementary Figure 3 (a) [7]. It is based on the optical intersubband transition (ISBT). Take the conduction band (CB) as an example, ISBT refers to the electronic transition in confined state as shown by an arrow. Because of the selection rule of ISBT, a normal incidence geometry (i.e., light incident normal to the wafer and along the growth direction) is not suitable. A commonly employed 45° edge facet geometry is shown Supplementary Figure 3 (c). This geometry “throws away” one half of the unpolarized light, but is simple and convenient to realize, and is usually used to obtain a detector performance benchmark [8]. The 45° edge facet geometry is not suitable for practical application, because it will cause the image stretched and distorted. There must be extra optical coupling structure to realize the normal incidence absorption. The most commonly used is the diffractive grating structure, which adds extra cost and increases difficulty of fabrication.

The basic structure unit of HIWIP detector consists of a heavily doped emitter layer and an intrinsic layer, as depicted in Supplementary Figure 3 (b). The doping concentration of the emitter layer is above the Mott transition. Due to the heavy doping level, the valance band edge of the emitter layer is always lower than that of the intrinsic layer because of band-gap narrowing effect. The Fermi

level is above the valence band edge of the emitter layer but still below that of i layer (intrinsic layer). The detection mechanism involves the free carrier absorption in highly doped emitter layers, followed by the internal photoemission of photoexcited carriers across the junction barrier and then collection under the bias[9].

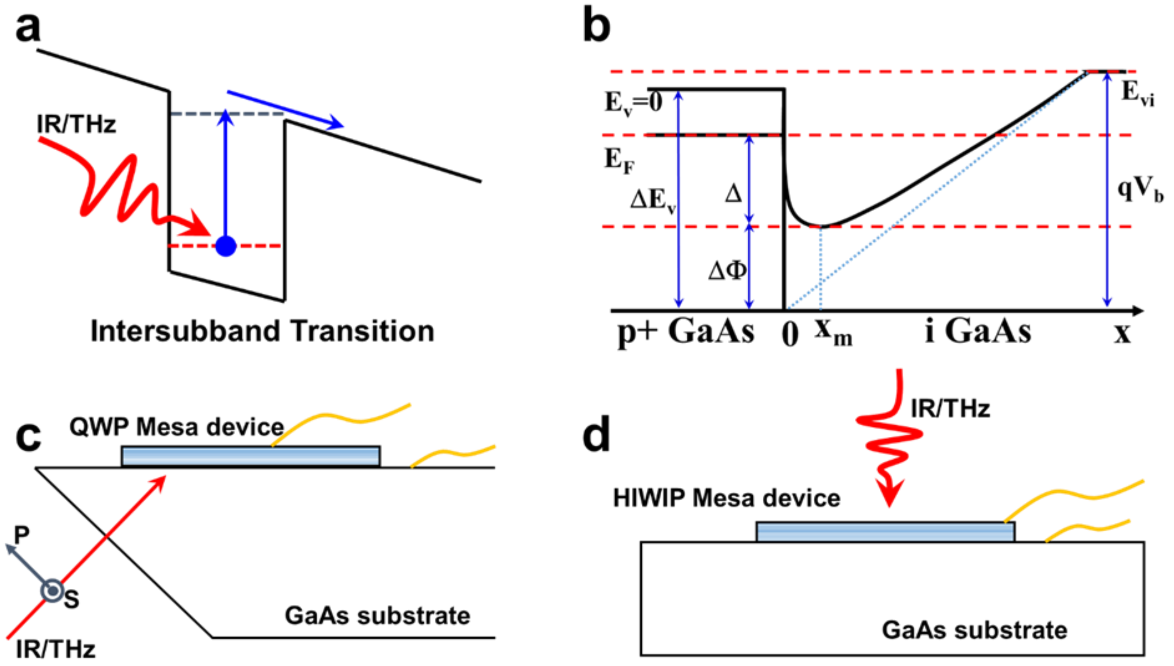

Supplementary Figure 3. The comparison of the quantum well photodetector (QWP) and homojunction interfacial workfunction internal photoemission (HIWIP) detector. (a) Intersubband transition in the QWP, (b) the homojunction interfacial workfunction internal photoemission detection mechanism, (c) 45° edge facet coupling geometry of QWP, (d) normal incident geometry of HIWIP.

Like most of the optoelectronic photodetectors, the range of the spectral response is restricted by the activation energy (Δ) [10]. The cutoff wavelength is dominated by the spectral rule ($\lambda_c(\mu m) = 1.24 / \Delta(eV)$), where Δ is the interfacial workfunction between the emitter layer and the intrinsic layer mainly determined by the doping concentration and applied bias. According to previous studies, the tailorable cutoff wavelength of the p-GaAs HIWIP detector could be realized by adjusting the doping level of the emitter layer between the Mott transition value and the critical value [11]. Because there is no the selection rule restriction, normal incident is permitted (show in Supplementary Figure 3 (d)).

Currently, the responsivity of an optimized single HIWIP detector optimized aiming below the frequency of Reststrahlen band could reach as high as 6.8 A/ W (corresponds to the quantum efficiency of 14.1%) at 5 THz [9], which is higher than that of a QWP with a complicated patch antenna (~5 A/W (corresponds to the quantum efficiency of 10.36 %) at 5 THz [12]). For the optimized HIWIP, the NEP could also reach as low as 2.3 pW/Hz$^{1/2}$. And minimum NEP of the patch antenna QWP ~10 pW/Hz$^{1/2}$ was realized.

The most important advantage is that the HIWIP allows normal incidence excitation thus bypassing the need for a grating coupler required for n-QWP device, which will reduce the cost, simplify the fabrication and realize high responsivity. Additionally, the QWP is a narrow band detector, the optical coupler design and realization is a great challenge when it was used for multicolor imaging. In contrast, the HIWIP allows normal incidence and shows a broadband photo-response over a wide frequency range, which may be more promising for multicolor imaging application.

Supplementary Note 5: Wafer details of the sample

p-GaAsTHz HIWIP-LED							
**Layer ID**	**Period**	**Material**	**Composition**	**Thickness(Å)**	**Dopant**	**Doping type**	**Doping (cm^{-3})**
11	--	GaAs	--	500	Si	n	2.50E+18
10	--	AlxGa1-xAs	x=0.02 >0.10	800	Si	n	2.50E+18
9	--	GaAs	--	400			
8	--	InyGa1-yAs	y=0.10	90			
7	--	GaAs	--	400			
6	--	AlxGa1-xAs	x=0.02	800			
5	20	GaAs	--	150	Be	p	8.00E+18
4	20	GaAs	--	800			
3	--	GaAs	--	1000	Be	p	8.00E+18
2	--	GaAs	--	3000	Be	p	3.00E+18
1	--	GaAs	--	200			
4-inch, GaAs(100) 625μm, semi-insulating substrate							

Supplementary Table 1. The wafer details of the integrated homojunction interfacial workfunction internal photoemission (HIWIP) detector and LED.

Supplementary References:

[1]. Schubert, E. F. Light-emitting diodes. (Cambridge University Press, 2006)

[2]. Ryu, H. Y., Kim, H. S., Shim, J. I. Rate equation analysis of efficiency droop in InGaN light-

emitting diodes. *Appl. Phys. Lett.* **95**, 081114 (2009)

[3]. Zhang, S., Wang, T. M., Hao, M. R., Yang, Y., Zhang, Y. H.et al. Terahertz quantum-well photodetectors: Design, performance, and improvements. *J. Appl. Phys.* **114**, 194507 (2013).

[4]. Ryzhii, V., Liu, H. C., Khmyrova, I., Ryzhii, M. Analysis of integrated quantum-well infrared photodetector and light-emitting diode for implementing pixelless imaging devices. *IEEE J. Quantum Electron.* **33**, 1527-1531 (1997).

[5]. Tsutsui, N., Khmyrova, I., Ryzhii, V., & Ikegami, T. Effect of photon recycling in pixelless imaging device. *Jpn. J. Appl. Phys.* **39** 5080 (2000).

[6]. Schneider, H., Liu, H. C. *Quantum Well Infrared Detectors* pp 15. (Springer, Berlin, 2007).

[7]. Schneider, H., Liu, H. C. *Quantum Well Infrared Detectors* pp 14. (Springer, Berlin, 2007).

[8]. Bai, P., Zhang, Y. H., Guo, X. G., Fu, Z. L., Cao, J. C., & Shen, W. Z. Realization of the high-performance THz GaAs homojunction detector below the frequency of Reststrahlen band. *Appl. Phys. Lett.* **113**, 241102 (2018).

[9]. Shen, W., Perera, A. U., Francombe, M. H., Liu, H. C., Buchanan, M., Schaff, W. J. Effect of emitter layer concentration on the performance of GaAs p-i homojunction far-infrared detectors: a comparison of theory and experiment. *IEEE T. Electron. Dev.* **45**, 1671-1677. (1998).

[10]. Shen, W. Z., Perera, A. G. U., Liu, H. C., Buchanan, M., Schaff, W. J. Bias effects in high performance GaAs homojunction far-infrared detectors. *Appl. Phys. Lett.* **71**, 2677-2679 (1997).

[11]. Palaferri, D. et al. Patch antenna terahertz photodetectors. *Appl. Phys. Lett.* **106**, 161102 (2015).

[12]. Bai, P., Zhang, Y. H., Shen, W. Z. Infrared single photon detector based on optical up-converter at 1550 nm. Sci. Rep. 7(1), 15341 (2017).

[13]. Wang, L. et al. Semiconductor up-converter based on cascade carrier transport for infrared detection/imaging. Appl. Phys. Lett. 107(13), 131107 (2015).
